# Nonalcoholic Fatty Liver Disease in Shift Workers and Its Effect on Peripheral Nerve Conduction: A Cross-Sectional Study

**DOI:** 10.7759/cureus.60632

**Published:** 2024-05-19

**Authors:** Dipali K Chatur, Saroj K Pati, Jayshri R Ghate, Rachita Nanda, Meenakshi Sinha, Kalpana Kodapi

**Affiliations:** 1 Physiology, All India Institute of Medical Sciences, Raipur, Raipur, IND; 2 Radiodiagnosis, All India Institute of Medical Sciences, Raipur, Raipur, IND; 3 Biochemistry, All India Institute of Medical Sciences, Raipur, Raipur, IND; 4 Pathology, All India Institute of Medical Sciences, Raipur, Raipur, IND

**Keywords:** peripheral nerves, metabolic disorder, shift workers, nerve conduction study, nafld

## Abstract

Introduction

Nonalcoholic fatty liver disease (NAFLD) presents as a multisystem disorder, heightening the risk of developing type 2 diabetes mellitus (T2DM) and cardiovascular diseases (CVDs). Occupation emerges as a significant factor influencing the occurrence of NAFLD. Research indicates that individuals engaged in shift work face an elevated risk of NAFLD, alongside obesity and T2DM, attributed to disruptions in their circadian rhythm, which precipitate hepatic steatosis and inflammation. Remarkably, peripheral neuropathy has been observed in conjunction with advanced liver disorders and NAFLD in the general population. However, the correlation between NAFLD and peripheral neuropathy remains unestablished in shift workers.

Objective

To identify NAFLD in seemingly healthy rotating shift workers and assess any potential impact of NAFLD on nerve function in this demographic.

Methods

This cross-sectional study involved 73 apparently healthy nonalcoholic security guards (aged 35 to 60 years) working in rotating shifts. The study included a comprehensive assessment, beginning with a medical history, an evaluation of physical activity, and anthropometric measurements. Confirmation of NAFLD was achieved through abdominal ultrasonography (USG), followed by the analysis of biochemical parameters. Motor and sensory nerve conduction studies (NCS) were conducted on participants with normal vitamin B12 levels using the Aleron electromyograph (EMG) machine (Recorders and Medicare Systems Private Ltd, Budanpur, India). The evaluation encompassed the Median and Common Peroneal motor nerves, as well as Median and Sural sensory nerves. Recorded parameters for motor nerves included distal motor latency (DML), compound muscle action potential (CMAP) amplitude, conduction velocity (CV), and F-wave minimum latency (F-wave), while sensory nerve parameters comprised sensory onset latency (SOL), sensory nerve action potential (SNAP) amplitude, and CV.

Results

Among 73 healthy security guards working in rotating shifts, 76.1% were diagnosed with NAFLD through abdominal ultrasound. Following participant withdrawals and exclusions due to vitamin B12 deficiency, a comparison of NCS parameters between NAFLD (n=24) and Non-NAFLD (n=12) groups revealed no significant disparities in motor or sensory parameters, except for a slightly diminished CMAP amplitude in the peroneal nerve of NAFLD subjects (8.21±2.83mV vs ±10.22±2.30 mV, p< 0.040). However, these differences fell within normal ranges, indicating no notable impact on peripheral nerve conduction in the presence of NAFLD.

Conclusion

The results indicate a high prevalence of NAFLD among individuals working rotating shifts. Moreover, the investigation suggests that despite the presence of NAFLD, there is no discernible influence on motor and sensory peripheral nerve conduction, particularly in common peroneal, median, and sural nerves.

## Introduction

Nonalcoholic fatty liver disease (NAFLD) is characterized by fat accumulation in the liver in individuals without excessive alcohol consumption. NAFLD ranges from simple steatosis to more severe conditions such as nonalcoholic steatohepatitis (NASH), which can lead to liver cirrhosis, fibrosis, and hepatocellular carcinoma [1,2]. Its prevalence has been increasing globally, estimated at 29.84% according to recent studies, [3], with varying rates in different populations, such as India where it ranges from 9% to 32% [2]. NAFLD is commonly associated with obesity, sedentary lifestyle, dyslipidemia, and metabolic syndrome [4]. However, research suggests that around 40% of the global NAFLD population is nonobese, with a significant proportion being lean-built individuals, emphasizing the need for broader screening criteria beyond obesity [5]. NAFLD is considered a multisystem disorder, increasing the risk of developing type 2 diabetes mellitus (T2DM), cardiovascular diseases (CVDs), and chronic kidney disease [6,7].

Various studies have suggested for association between the late stages of NAFLD and peripheral neuropathy, a condition characterized by nerve damage in the peripheral nervous system. Peripheral neuropathy is challenging to treat and can significantly impact a person's quality of life, increasing the risk of ulcers and even amputation. Studies by Chaudhry et al. in 1999 conducted prospective studies on patients with chronic liver disease, revealing a high prevalence of peripheral neuropathy, with sensorimotor neuropathy observed in 71% of patients and autonomic neuropathy in 48% [8]. Notably, 10.34% of patients with liver cirrhosis and neuropathy had no known etiology, indicating a cryptogenic origin in some cases. Similar findings were reported in the Indian population by Kharbanda et al. in 2003, where nerve conduction studies (NCS) showed abnormalities in 73% of patients, predominantly manifesting as axonal sensory-motor polyneuropathy [9]. The study suggested that liver disease itself, regardless of its etiology, could be a probable cause of neuropathy in these patients.

Considering the greater prevalence of cryptogenic neuropathies of chronic liver disorder and liver cirrhosis, alteration in nerve conduction parameters can occur at earlier stages also. Huang et al. 2021 assessed the correlation of diabetic peripheral neuropathy (DPN) with liver steatosis and fibrosis [10]. They conducted an electrodiagnostic nerve conduction study and FibroScan for liver steatosis and fibrosis. Their findings revealed a heightened prevalence of DPN, with liver fibrosis identified as independently associated with an elevated risk of DPN. However, the literature contains limited reports on the association between NAFLD and polyneuropathy.

Large fiber neuropathy is characterized by the loss of joint position and vibration sense, leading to sensory ataxia. Conversely, small fiber neuropathy presents with impaired pain, temperature perception, and autonomic functions [11]. Recently, Gu et al. 2023 reported a significant association and concluded that the small nerve fibers get affected in NAFLD [12]. They used the US fatty liver index (FLI) score for NAFLD detection and the monofilament test for the assessment of peripheral neuropathy. However, the link between NAFLD and peripheral neuropathy is still unclear, except for a few reports on the diabetic population and a single study with a subjective evaluation of peripheral neuropathy in the general population. Additionally, to the best of our knowledge, there is no study assessing large nerve fiber damage using NCS in the normal population.

In this context, another demographic at heightened risk for metabolic disorders is shift workers, who are considered part of the healthy population. Approximately one-fifth of the global population engages in shift work [13,14], with shift workers experiencing high levels of occupational stress compared to daytime workers [15-18]. Studies indicate that shift workers, especially those on rotating shifts, have a higher risk of developing NAFLD because of disruptions in their circadian rhythm, leading to hepatic steatosis and inflammation [19]. Peripheral nerve damage is commonly recognized clinically and reported to be associated with NAFLD. However, objectively, the impact on larger nerve fibers remains largely uninvestigated at the earlier stage of NAFLD, particularly in shift workers, who exhibit a high prevalence of NAFLD. Moreover, given the reported prevalence of metabolic disorders in shift workers, there may be an increased likelihood of developing neuropathy.

Therefore, this study was planned to identify NAFLD in seemingly healthy rotating shift workers and assess any potential impact of NAFLD on nerve function in this demographic.

## Materials and methods

Study design

This cross-sectional observational analytical type of study was conducted at the Department of Physiology in collaboration with the Department of Radiodiagnosis and the Department of Biochemistry at AIIMS Raipur, Chhattisgarh.

Sample size

The sample size was determined by the formula n=z2pq/e2 for a cross-sectional study with a prevalence (p) of 22% [2]. The calculated sample size was 65.89. Considering the 10% rejection rate, it was rounded off to 73.

Ethical consideration

This study was started after obtaining ethical approval from the Institutional Ethics Committee of AIIMS Raipur, Chhattisgarh (Letter No: 2153/IEC-AIIMSRPR/2022 dated 28.02.2022).

Study criteria

This study was performed on apparently healthy security guards involved in rotating shift work in the institute. The study included a total of 73 participants of both sexes, aged between 35 and 50 years, who were nonalcoholic [20], had no health issues in the past six months, and were willing to participate. Participants having a history of diabetes, hypertension, and CVDs; a history of known liver (hepatitis B and C) and kidney disorders; severe bleeding tendency; cancer; a history of psychiatric illness; eating disorders; and smokers of more than one pack year [21] were excluded from the study. Subjects taking drugs for immune suppression or modulation of metabolism, being pregnant or having breastfed within the past 12 months, history of myopathy and trauma, skin lesion, or swelling during nerve that interferes with NCS recordings were also excluded.

NAFLD was diagnosed and graded by a radiologist using an ultrasonography (USG) machine, WIPRO GE LOGIQ S8 (Wipro GE Healthcare Pvt. Ltd., Bengaluru, India) with a curvilinear probe (3.5-5 MHz). The grading of fatty infiltration of the liver was recorded as grades 0, 1, 2, and 3. Grade 0: Normal echogenicity of the right liver lobe in comparison with the cortex of the right kidney; Grade 1: slight, diffuse increase in fine echoes in liver parenchyma with normal visualization of diaphragm and intrahepatic vessel borders; Grade 2: moderate, diffuse increase in fine echoes with slightly impaired visualization of intrahepatic vessels and diaphragm; Grade 3: marked increase in fine echoes with poor or non-visualization of the intrahepatic vessel borders, diaphragm, and posterior right lobe of the liver [22].

Procedure

A total of 73 security guards working in rotating shifts were recruited based on our inclusion and exclusion criteria. An informed written consent was taken from each participant. After obtaining a medical history and conducting a general examination, their weights and heights were recorded to calculate BMI using the formula of weight in kilograms divided by height in meters squared. Physical activity levels were assessed using the short form of the International Physical Activity Questionnaire (IPAQ-SF), categorizing activity levels as inactive, minimally active, and health-enhancing physical activity (HEPA) [23]. The presence of NAFLD was confirmed through abdominal (USG performed by a radiologist. Blood samples were drawn to estimate serum vitamin B12 levels. Following this, a few participants were excluded based on B12 deficiency (13 NAFLD and three non-NAFLD), while a few subjects withdrew from the study before the NCS.

Following this, NCS were conducted on the dominant side of participants using the Aleron electromyograph (EMG) machine (Recorders and Medicare Systems Pvt. Limited (RMS), Recorders and Medicare Systems Private Ltd., Budanpur, India) at room temperature of 22°C to 26°C. The median and common peroneal motor nerves, as well as median and sural sensory nerves, were recorded. The filter settings for sensory nerve conduction were 5-10 Hz (low frequency), 2-3 KHz (high frequency) with gain/sensitivity of 1-5 μV/mm, and sweep speed of 1-2 ms/mm. The filter settings for motor nerve conduction were 5-10 Hz (low frequency), 10-12 KHz (high frequency) with gain/sensitivity of 1-5 mV/mm, and sweep speed: 2-5 ms/mm. The stimulus used was a square pulse of 0.1 ms duration with an intensity of 5-40 mA. The signals were averaged for the sensory conduction study.

Motor nerve parameters included distal motor latency (DML) in milliseconds, compound muscle action potential (CMAP) amplitude in millivolts, conduction velocity (CV) in meters per second, and the late response (i.e., F-wave minimum latency (F min latency) in milliseconds). For sensory nerves, sensory nerve action potential (SNAP) amplitudes in microvolts and CV in meters per second were recorded.

Assessment of NCS

We adopted standard procedures for motor and sensory conduction studies given by Misra et al. [24].

Common peroneal motor nerve: Belly tendon montage was used with a recording electrode placed on the extensor digitorum brevis muscle and reference electrode distally over the metatarsal-phalangeal joint of the little toe. The peroneal nerve was stimulated at the ankle slightly lateral to the tibialis anterior tendon (distal stimulation) and one to two finger breadths inferior to the fibular head on the lateral calf (proximal stimulation).

Median motor nerve: An active electrode was placed over the abductor pollicis brevis muscle belly, while a reference electrode was placed over the first metacarpal-phalangeal joint. A median nerve was stimulated at the middle of the wrist between the tendon of flexor carpi radialis and palmaris longus and proximally stimulated over the brachial artery pulse at the antecubital fossa.

F-wave of the common peroneal and median nerve: In the motor conduction study using the same electrode settings, the recording of F-waves was conducted using supramaximal stimulation.

Sural nerve: A surface electrode was positioned posterior to the lateral malleolus, while a reference electrode was placed 3-4 cm distally. The sural nerve was stimulated antidromically at the posterior-lateral calf 14 cm proximal to the recording electrode.

Median sensory nerve: The recording site for the study was the index or middle finger (digits 2 and 3), and ring electrodes were utilized with the active electrode positioned over the metacarpal-phalangeal joint. Additionally, the reference electrode was placed 3-4 cm distally over the distal interphalangeal joint. The middle of the wrist between the tendons to the flexor carpi radialis and palmaris longus was stimulated at approximately 13 cm distally.

Statistical analysis: The statistical analysis was conducted using Statistical Product and Service Solutions (SPSS; IBM SPSS Statistics for Windows, Armonk, NY), with data expressed as mean and standard deviation. An unpaired t-test was utilized to calculate differences in mean values between the two groups. A P value of less than 0.05 was considered statistically significant. A data analysis for NCS was performed for participants with normal vitamin B12 levels after excluding those with vitamin B12 deficiency [25].

## Results

**Figure 1:**
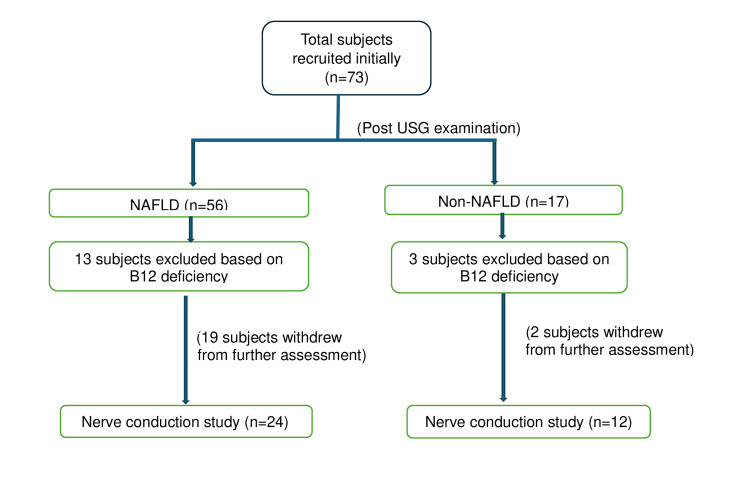
Recruitment of participants in the study for screening and nerve conduction study

A total of 73 apparently healthy security guards (47 male and 26 female), who were willing to participate and met the inclusion criteria, were recruited for the study. They underwent anthropometric measurement, physical activity level assessment, and abdominal USG for detection of NAFLD. Afterward, only a few participants left the study, and a few were excluded based on B12 deficiency before their assessment for NCS (Figure 1).

The duration of shift work of all the participants, on average, was 9.31±4.73 years. The mean age of the participants was 40.04±4.68 years, and the mean BMI was 25.29±3.50 kg/m^2^. Based on abdominal USG, 76.1% (56) of the participants were confirmed to have NAFLD, while 24.9% (17) were classified as non-NAFLD. Most of the participants had NAFLD grade I (55%) and grade II (45%) based on USG findings. A comparison of demographic and anthropometric data of the studied groups showed no significant difference in the age of the participants; however, BMI was significantly higher in the NAFLD group (Table 1).

**Table 1:** Comparison of demographic and anthropometric data of NAFLD and non-NAFLD shift workers *A P value of <0.05 is considered statistically significant. NAFLD: Nonalcoholic Fatty Liver Disease; M: Male; F: Female; BMI: body mass index

	NAFLD (n= 56)	Non-NAFLD (n= 17)	P value
Age (yrs)	40.59±4.55	39.19±4.82	0.294
M:F	40:16	7:10	
BMI (kg/m^2^)	25.54±3.43	23.88±2.77	0.045 *

The assessment of physical activity, as determined by IPAQ-SF, revealed that the energy expenditure in MET-min/week was higher in the NAFLD group compared to the non-NAFLD group (2154.45±2967.84 vs 2014.58±2482.42, p=0.966). Moreover, the sitting time per week was greater in the NAFLD group, although this difference was not statistically significant (182.85±135.40 vs 150.58±102.84, p=0.505).

In the NAFLD group, 37.5% of the 56 participants were inactive, 37.5% were minimally active, and 25% were engaging in health-enhancing physical activity. In contrast, the non-NAFLD group had 23.53% inactive participants, 52.94% minimally active, and 23.52% engaged in health-enhancing physical activity.

NCS parameters of participants

The nerve conduction study was performed on 24 participants of the NAFLD group and 12 participants of the non-NAFLD group as depicted in Figure 1.

Motor Nerve Conduction Study of the Peroneal and Median Nerve

In the NAFLD group, the CMAP amplitude of the peroneal nerve was significantly lower compared to the non-NAFLD group (7.97±2.84 vs 10.22±2.30, p=0.022). However, the slightly reduced CMAP amplitude of the median nerve and CV of both the nerves along with prolongation of DML and F-min latency was recorded, which were statistically not significant (Table 2).

**Table 2:** Comparison of mean value of motor nerve conduction study in NAFLD and non-NAFLD shift workers group *A P value of <0.05 is considered statistically significant. NAFLD: nonalcoholic fatty liver disease; CMAP: compound muscle action potential

Type of nerve	NAFLD (n= 24)	Non-NAFLD (n= 12)	P value
Median motor nerve			
Distal motor latency (ms)	3.49±0.46	3.31±0.48	0.297
CMAP amplitude (mV)	15.58±3.06	16.87±5.30	0.363
Conduction velocity (m/sec)	55.78±5.17	55.84±4.10	0.971
F-min latency (ms)	26.5±1.66	25.45±2.83	0.169
Peroneal motor nerve			
Distal motor latency (ms)	3.94±0.67	3.67±0.57	0.242
CMAP amplitude (mV)	8.21±2.83	10.22±2.30	0.040*
Conduction velocity (m/sec)	50.53±3.42	50.53±3.71	0.828
F-min latency (ms)	47.19±4.46	45.35±2.82	0.224

Sensory Nerve Conduction Study of the Sural and Median Nerve

In the NAFLD group, the SNAP amplitude of the sural and median nerves was slightly lower compared to the non-NAFLD group with no statistical significance. Additionally, the CV of the sural nerve was slightly less, and the median nerve slightly increased without any statistical significance (Table 3).

**Table 3:** Comparison of mean value of sensory nerve conduction study in NAFLD and non-NAFLD shift workers group A P value of <0.05 is considered statistically significant. NAFLD: nonalcoholic fatty liver disease, SNAP: sensory nerve action potential

Type of nerve	NAFLD (n=24)	Non-NAFLD (n=12)	P value
Median sensory nerve			
SNAP amplitude (µV)	48.43±14.10	49.28±21.32	0.889
Conduction velocity (m/sec)	47.25±5.12	45.31±5.38	0.302
Sural nerve			
SNAP amplitude (µV)	15.90±10.69	17.78±10.29	0.621
Conduction velocity (m/sec)	45.02±6.53	46.08±2.57	0.595

## Discussion

In our study, we found a high prevalence of NAFLD among security guards working in rotating shifts, with 76.1% affected. This prevalence is notably higher compared to similar studies conducted by Kiseok et al. in 2022 (44.5%) and Zhang et al. in 2020 (33.7%) among shift workers in steel plants in Korea and China, respectively [26,19]. The latest prospective analysis of 281,280 UK Biobank participants reports that workers who occasionally worked night shifts were 1.12 times more and individuals with usual or permanent night shifts had a 1.27 times higher risk of developing (NAFLD), and this association remained significant irrespective of genetic predisposition to NAFLD, indicating that night shift work is associated with an increased risk of incident of NAFLD [27]. Rotating shifts disturb melatonin hormone secretion thereby disrupting circadian rhythm, which can affect the metabolic homeostasis of the liver. This can result in an imbalance in the use and accumulation of nutrients through bile acid degeneration [26]. The deranged balance of carbohydrate and fat metabolism causes fat to build up in the liver, which leads to NAFLD. Alongside these findings, the potential mechanism underlying NAFLD may involve reduced protective function of melatonin against oxidative stress, as suggested by Kim et al. 2022 [26], as decreased levels of melatonin have been observed in shift workers [19].

Physical activity levels were assessed using the IPAQ-SF questionnaire, revealing greater sitting time among individuals in the NAFLD group compared to those in the non-NAFLD group. Most participants in both groups were found to be inactive. Additionally, the NAFLD group exhibited a significantly higher BMI compared to the non-NAFLD group. According to BMI criteria for the Asian population, the NAFLD group fell into the obese category, whereas the non-NAFLD group was classified as overweight [28]. This aligns with similar findings reported by Zhang et al. in 2020, indicating higher BMI among NAFLD shift workers [19]. The NAFLD group, characterized by multiple risk factors such as increased sitting time and higher BMI, may contribute to the elevated prevalence of NAFLD observed in this study.

Before analyzing NCS data, participants with vitamin B12 deficiency were excluded because of its adverse effects on NCS parameters [29]. Our results regarding NCS of large motor and sensory nerves exhibited variability. We observed a decrease in the CMAP amplitude of the common peroneal and median motor nerves as well as the sensory nerve action potential (SNAP) amplitude of the sural sensory nerve; however, the decrease was significant only in the common peroneal nerve. Similarly, changes in the CV of common peroneal, median motor, sural sensory nerves, and median sensory nerve were statistically nonsignificant.

On average, the peroneal motor nerve is commonly affected by various factors such as compression [30] or habitual leg-crossing [31]. Reduced nerve conduction amplitude with normal nerve CV may indicate a dropout of smaller-diameter axons, resulting in decreased CMAP amplitude while maintaining normal CV. Therefore, changes in the common peroneal nerve alone may not confirm neuropathy. Additionally, all observed changes in nerve conduction study parameters were within normal limits, making it difficult to conclude them as neuropathic changes. NCS is considered an objective, reliable, and reproducible technique, serving as the gold standard for neuropathy detection. It primarily detects the involvement of large nerve fibers, and, based on our results, there may not be significant involvement of large nerve fibers in NAFLD [24].

Gu et al. reported a significant association between NAFLD and peripheral neuropathy in the general population aged 40-64 years, both in nondiabetic and diabetic individuals [12]. They utilized the US FLI score for NAFLD detection and conducted a monofilament assessment of neuropathy, a subjective test for peripheral neuropathy assessment. Their findings indicated the presence of small fiber neuropathy in individuals with NAFLD. Lv et al. reported a negative association between the prevalence of NAFLD and DPN in hospitalized Chinese individuals with T2DM [32]. However, Kim et al. 2014 found no association between NAFLD and DPN [33] in Korean individuals with T2DM based on NCS.

Limitations of the study

The prevalence mentioned in the study is based on a limited sample of rotating shift workers employed in a tertiary care center. Hence, results cannot be generalized to the normal population. The study utilized USG for the detection of NAFLD, but it acknowledged that elastography could provide a more comprehensive understanding. Furthermore, because of the inclusion of apparently healthy individuals, there was a reluctance to undergo NCS, highlighting a limitation in data collection. Therefore, more extensive studies with larger sample sizes are needed to confirm the findings and address these limitations effectively.

## Conclusions

Based on the above discussion, it is apparent that individuals employed in rotating shift work schedules exhibit a significant prevalence of NAFLD, despite presenting with an outward appearance of good health. Interestingly, although NAFLD prevails among these workers, there appears to be no observable impact on the conduction of peripheral nerve fibers. Consequently, it is recommended to implement regular screenings for NAFLD and other associated metabolic abnormalities within this demographic. By proactively addressing these health concerns, the potential development of liver diseases, diabetes, and cardiovascular conditions among rotating shift workers may be avoided, thereby promoting overall well-being and quality of life within this population segment.
